# Gene Dosage Analysis in a Clinical Environment: Gene-Targeted Microarrays as the Platform-of-Choice

**DOI:** 10.3390/microarrays2020051

**Published:** 2013-03-27

**Authors:** Renate Marquis-Nicholson, Debra Prosser, Jennifer M. Love, Donald R. Love

**Affiliations:** 1 Diagnostic Genetics, LabPLUS, Auckland City Hospital, P.O. Box 110031, Auckland 1148, New Zealand; E-Mails: renate.mn@gmail.com (R.M.-N.); DProsser@adhb.govt.nz (D.P.); JLove@adhb.govt.nz (J.M.L.); 2 School of Biological Sciences, University of Auckland, Private Bag 92019, Auckland 1142, New Zealand

**Keywords:** array comparative genomic hybridisation (aCGH), dosage analysis, targeted microarray, molecular diagnosis, maturity-onset diabetes of the young (MODY), familial phaeochromocytoma/paraganglioma syndrome, Cowden syndrome

## Abstract

The role of gene deletion and duplication in the aetiology of disease has become increasingly evident over the last decade. In addition to the classical deletion/duplication disorders diagnosed using molecular techniques, such as Duchenne Muscular Dystrophy and Charcot-Marie-Tooth Neuropathy Type 1A, the significance of partial or whole gene deletions in the pathogenesis of a large number single-gene disorders is becoming more apparent. A variety of dosage analysis methods are available to the diagnostic laboratory but the widespread application of many of these techniques is limited by the expense of the kits/reagents and restrictive targeting to a particular gene or portion of a gene. These limitations are particularly important in the context of a small diagnostic laboratory with modest sample throughput. We have developed a gene-targeted, custom-designed comparative genomic hybridisation (CGH) array that allows twelve clinical samples to be interrogated simultaneously for exonic deletions/duplications within any gene (or panel of genes) on the array. We report here on the use of the array in the analysis of a series of clinical samples processed by our laboratory over a twelve-month period. The array has proven itself to be robust, flexible and highly suited to the diagnostic environment.

## 1. Introduction

Large deletions and duplications have long been recognised as playing an important part in the aetiology of several disorders conventionally diagnosed using molecular techniques, such as Duchenne Muscular Dystrophy (DMD) and Charcot-Marie-Tooth Neuropathy Type 1A (CMT1A) [1,2]. In addition to these classical deletion/duplication disorders, the role of partial or whole gene deletions in the pathogenesis of a wide variety of single-gene disorders is becoming increasingly evident. A 2008 review of the entries in the online Human Gene Mutation Database showed that large deletions and duplications comprise 10% of the listed mutations [3], compared to 6% in 2003 [4]. This number will continue to rise as the increasingly widespread availability of cost-effective and robust analysis techniques enables more individuals to be subjected to dosage analysis on a routine basis.

A variety of dosage analysis methods are available to the molecular diagnostic laboratory, including multiplex ligation-dependent probe amplification (MLPA) [5], quantitative real-time PCR (qPCR) [6] and customised fluorescence *in situ* hybridisation (FISH) [7]. Each of these methods, however, is relatively expensive and, in the case of MLPA and qPCR, is confined to a limited number of exons across a limited number of genes [8,9]. Low sample throughput in a small diagnostic laboratory prevents batching of samples if turn-around times are to be maintained, thereby further decreasing the cost-effectiveness of assays with such a limited scope. It can also be difficult to maintain staff competency across the full range of dosage assays required when sample numbers are modest.

In order to address these limitations we have implemented the use of a bespoke Nimblegen 12 × 135 K CGH Array. This array targets a panel of genes chosen to complement the sequencing assays we offer in-house, as well as covering a number of genes (such as *PMP22*) for which partial or whole gene deletion/duplication is the predominant pathogenic mechanism. In addition to this gene-focused coverage, the array also provides low-density coverage of the entire human genome, which allows for carrier testing of genomic rearrangements that may have initially been detected by high density molecular karyotyping of a proband. 

We have previously reported on the validation of this custom-designed array and the cost-effectiveness of the method in a small diagnostic laboratory [10]. Here, we report on the use of the array in the routine investigation of a series of clinical samples that illustrate the suitability and flexibility of this approach for dosage analysis in a diagnostic environment.

## 2. Experimental Section

### 2.1. Patient Samples

Peripheral blood EDTA samples from ninety-eight individuals were submitted over a twelve-month period to the Diagnostic Genetics department of LabPLUS, Auckland City Hospital, for molecular analysis of a range of genes (see Table 1). An archived Guthrie card, collected as part of routine newborn screening, was retrieved for one additional (deceased) patient. Analysis was requested principally for diagnostic purposes (eighty patients), with the remaining samples received for either carrier or predictive testing. Dosage analysis was performed as the primary assay for the *PMP22* and *DMD* genes, as deletion/duplication is the predominant pathogenic mechanism in these genes [11,12,13]. Sequence analysis was performed first for the other genes, cascading to aCGH if no pathogenic mutations were found.

**Table 1:** Clinical samples analysed over a twelve-month period.

Gene(s) of interest	Number of patients	Clinical indication	Mode of inheritance	Sample type
*APC*	7	Familial adenomatous polyposis (FAP)	Autosomal dominant	Peripheral blood
Dystrophin ( *DMD*)	7	Becker muscular dystrophy (BMD)	X-linked	Peripheral blood
	17	Duchenne muscular dystrophy (DMD)		Peripheral blood; Guthrie spot (1)
	17	Carrier testing for BMD/DMD		Peripheral blood
Calcium-sensing receptor (*CaSR*)	1	Familial hypocalciuric hypercalcemia	Autosomal dominant	Peripheral blood
E-cadherin (*CDH1*)	5	Familial gastric cancer	Autosomal dominant	Peripheral blood
*EPCAM*	3	Familial colon cancer	Autosomal dominant	Peripheral blood
*HNF4α* (MODY1), *GCK* (MODY2), *HNF1α* (MODY3), *HNF1β* (MODY5)	3	Maturity-onset diabetes of the young (MODY); 1 individual also with hepatic multiple adenomatosis	Autosomal dominant	Peripheral blood
*PMP22*	19	Possible diagnosis of Charcot Marie Tooth Type 1A (CMT1A)	Autosomal dominant	Peripheral blood
	7	Possible diagnosis of Hereditary Neuropathy with liability to Pressure Palsies (HNPP)	Autosomal dominant	Peripheral blood
*MSH2*	2	Hereditary Non-Polyposis Colorectal Cancer (HNPCC)	Autosomal dominant	Peripheral blood
*PTEN*	3	Cowden syndrome	Autosomal dominant	Peripheral blood
*RET proto-oncogene, SDHAF2, SDHB, SDHC, SDHD, TMEM127, VHL*	6	Familial phaeochromocytoma/paraganglioma	Autosomal dominant	Peripheral blood
	1	Predictive testing for familial paraganglioma		
*VHL*	1	Possible diagnosis of Von-Hippel-Lindau syndrome	Autosomal dominant	Peripheral blood

### 2.2. DNA Extraction

Genomic DNA (gDNA) was extracted from peripheral blood EDTA samples using the Gentra Puregene DNA Extraction kit (Qiagen, Gaithersburg, MD, USA) and from the Guthrie card using the QIAmp DNA Miniblood Kit (Qiagen, Gaithersburg, MD, USA) as described by the manufacturer.

### 2.3. Dosage Analysis

A Roche NimbleGen 12 × 135 K custom CGH Array was used for dosage analysis. This bespoke CGH array was designed to interrogate the coding regions of sixty-six genes of interest to our laboratory. Exonic probes overlapped by 25 bp in order to provide high-resolution detection of deletions or duplications within the coding regions of the genes of interest. Intronic probes were spaced on average every 175 bp. In addition to the targeted probes, approximately 75,000 “backbone” probes, with a mean probe interval of 45 kbp, were also included, providing low-density whole genome coverage. 

Two hundred and fifty nanograms of gDNA were processed according to the manufacturer’s instructions; NimbleGen Array User’s Guide: CGH and CNV Arrays v6.0 [14]. In brief, extracted gDNA from samples and Promega controls was denatured in the presence of a Cy3-(test) or Cy5-(control) labelled random primers and incubated with the Klenow fragment of DNA polymerase, together with dNTPs (5 mM of each dNTP), at 37 °C for 2 h. The reaction was terminated by the addition of 0.5 M EDTA (21.5 µL), prior to isopropanol precipitation and ethanol washing. Following DNA quantitation, the test and sex-matched control samples were combined in equimolar amounts and applied to one of the twelve arrays on the microarray slide. Hybridisation was carried out in a Roche NimbleGen Hybridisation Chamber (Madison, WI, USA) for a period of 48 h. Slides were washed and scanned using a NimbleGen MS200 Microarray Scanner (Madison, WI, USA Array image files (.tif) produced by the MS200 Data Collection Software were imported into DEVA v1.2.1 (Roche NimbleGen Inc., Madison, WI, USA) for analysis. Data was filtered using a log_2_ratio threshold of less than −0.4 over 6 probes for a deletion and greater than 0.4 over 15 probes for a duplication. All copy number changes meeting these thresholds were exported out of DEVA into a Microsoft Excel spreadsheet for further investigation. Each genomic region exhibiting a copy number change within one of the genes of interest was examined using the UCSC genome browser [15] to determine the location and significance of the change. Analysis of copy number changes was only performed for the gene(s) of interest; changes identified within other genes for which analysis had not been requested were not subjected to detailed examination. 

## 3. Results and Discussion

Dosage changes were detected in twenty-six of the eighty patients referred in for diagnostic testing, nine of the seventeen referred in for carrier testing, and in the one patient referred in for predictive testing (see Table 2). These changes are separated into disease/gene and are described in detail below.

**Table 2:** Mutations detected by array comparative genomic hybridisation (aCGH) analysis of clinical samples.

Patient	Gene(s) analysed	Genotype	Significance of result
1,2	DMD	Hemizygous deletion of exons 45–47 (inclusive)	In-frame deletion; consistent with BMD phenotype
3		Hemizygous deletion of exons 45–48 (inclusive)	In-frame deletion; consistent with BMD phenotype
4		c.5199_5209del (p.Thr1734SerfsX10)	Premature truncation of protein; consistent with DMD phenotype
5		Hemizygous deletion of exons 46–50 (inclusive)	Out-of-frame deletion; consistent with DMD phenotype
6		Hemizygous duplication of exon 12	Out-of-frame duplication; consistent with DMD phenotype
7		Hemizygous duplication of exons 10–11 (inclusive)	Out-of-frame duplication; consistent with DMD phenotype
8		Hemizygous deletion of exons 53–59 (inclusive)	Out-of-frame deletion; consistent with DMD phenotype
9,10		Hemizygous duplication of exons 8–9 (inclusive)	Out-of-frame duplication; consistent with DMD phenotype
11–19		Various (heterozygous deletion/duplication)	Carrier of familial deletion/duplication
20	HNF1α	Heterozygous deletion of exons 2–3 (inclusive)	Consistent with clinical phenotype—adenomatosis and MODY3
21–29	PMP22	~1.5 Mb heterozygous duplication encompassing *PMP22* gene	Consistent with CMT1A phenotype
30,31		Reciprocal deletion	Consistent with HNPP phenotype
32,33	*PTEN*	Heterozygous deletion of exon 2	Consistent with Cowden syndrome phenotype
34,35	*SDHB*	Heterozygous deletion of exon 1	Consistent with clinical diagnosis of familial phaeo syndrome
36			Presence of familial deletion— appropriate surveillance/operative management required

### 3.1. PMP22 Gene Analysis—Charcot-Marie-Tooth Neuropathy Type 1A (CMT1A) and Hereditary Neuropathy with Liability to Pressure Palsies (HNPP)

**Figure 1:**
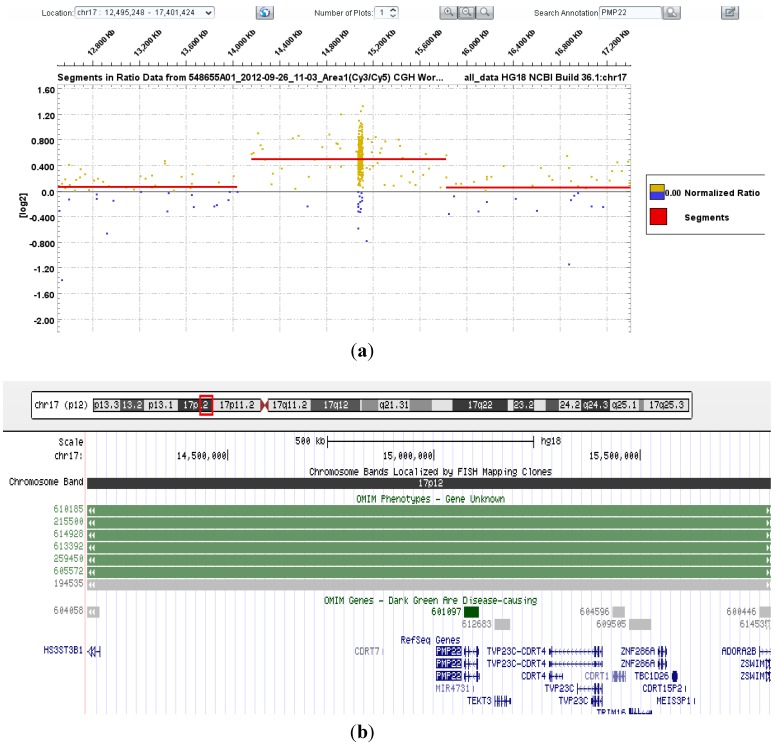
(**a**) DEVA software output showing copy number change (duplication; log2ratio: 0.4953) for probes localized to chr17: 14160052-15824662 (hg18 co-ordinates), encompassing the *PMP22* gene; (**b**) UCSC genome browser graphic output of chr17: 14160052-15824662 (hg18 co-ordinates).

Nineteen patients were referred for CMT1A gene analysis and seven for HNPP. Of these, two were found to carry the classic 1.5 Mb deletion (HNPP) and nine carried the reciprocal duplication (CMT1A) at 17p11.2 (includes the *PMP22* gene; see Figure 1) that is responsible for 80% of each of these disorders [11,12]. 

### 3.2. DMD Gene Analysis—Duchenne and Becker Muscular Dystrophy (DMD/BMD)

More than 5,000 mutations have been identified in individuals with BMD or DMD [13,16]. These mutations are highly variable and run the full spectrum from deletion of the entire gene, to deletion or duplication of one or more exons, to small deletions or insertions, to single-base pair alterations. Deletions and duplications account for 60–70% of cases of DMD and 5–10% of cases of BMD [17]. For this reason, deletion/duplication analysis is the first-line diagnostic test for DMD/BMD, with sequence analysis performed if no dosage changes are found. As a general rule, mutations that alter the reading frame correlate with DMD, whereas those that preserve the reading frame are associated with BMD [16,18]. 

Twenty-four males with a clinical diagnosis of dystrophinopathy were referred for routine diagnostic testing. Array CGH analysis revealed a hemizygous deletion or duplication within the *DMD* gene in ten of these patients. Further assessment of each of these mutations was performed using a Reading-frame Checker [19]. In each case the predicted effect was consistent with the phenotype that was observed clinically. An intra-exonic deletion of six probes, the lower limit of size threshold for analysis, was identified within exon 37 in Patient 4. Sequence analysis of exon 37 confirmed a hemizygous deletion of 11 base pairs within the exon, c.5199_5209del (p.Thr1734SerfsX10). This frameshift mutation results in premature termination of translation and truncation of the protein and is therefore consistent with the clinical diagnosis of DMD.

Molecular testing for BMD/DMD is not only useful to confirm the clinical diagnosis in affected males who are suspected to have a dystrophinopathy based on clinical signs and an elevated serum creatine kinase (CK) level, but identification of the causative mutation also informs genetic counselling for the family and allows carrier and prenatal testing to be performed as appropriate [20]. The familial mutation was identified in nine of the seventeen patients referred for *DMD* carrier testing during this twelve-month period. The absence of the familial mutation within female relatives within the extended family is reassuring, but lack of the familial mutation in the mother of an affected boy does not mean that the mutation is necessarily *de novo* as germline mosaicism remains a possibility. 

### 3.3. PTEN Gene Analysis

Three patients were referred for *PTEN* gene dosage analysis following a negative result on sequencing. Each of these patients had a probable clinical diagnosis of Cowden Syndrome, a multiple hamartoma syndrome that confers a high risk of benign and malignant tumours of the thyroid, breast, and endometrium [21]. Two of these three patients were found to carry a deletion encompassing exon 2 of the PTEN gene. Exonic or whole gene deletions are believed to be responsible for up to 10% of cases of Cowden syndrome [21,22]. The deletion of exon 2 is an out-of-frame deletion that alters the translational reading frame and results in premature truncation of the PTEN protein. It is extremely likely, therefore, to be the causative mutation in these cases. 

### 3.4. Familial Paraganglioma/Phaeochromocytoma Syndrome Mutation Screening—SDHAF2, SHDB, SDHC, SDHD, VHL, RET Proto-Oncogene, and TMEM127 Gene Analysis

The full familial paraganglioma/phaeochromocytoma gene panel (genes listed above) was analysed in six patients using both sequencing and aCGH. No pathogenic mutations were detected on sequence analysis in any of the genes for any of these patients. Array CGH revealed a deletion of exon 1 of the *SDHB* gene in two individuals. This deletion was later detected in the unaffected son of one of these patients. Heterozygous deletion of exon 1 of the *SDHB* gene has been reported in several unrelated families with hereditary phaeochromocytoma [23,24]. It has been proposed that the relatively high frequency of this deletion (three of the five instances of gross deletion listed in the online Human Gene Mutation Database) is due to a high density of Alu repeats within intron 1 of the *SDHB* gene [24]. 

### 3.5. Maturity-Onset Diabetes of the Young (MODY) Mutation Screening—HNF4α, GCK, HNF1α, HNF1β

**Figure 2:**
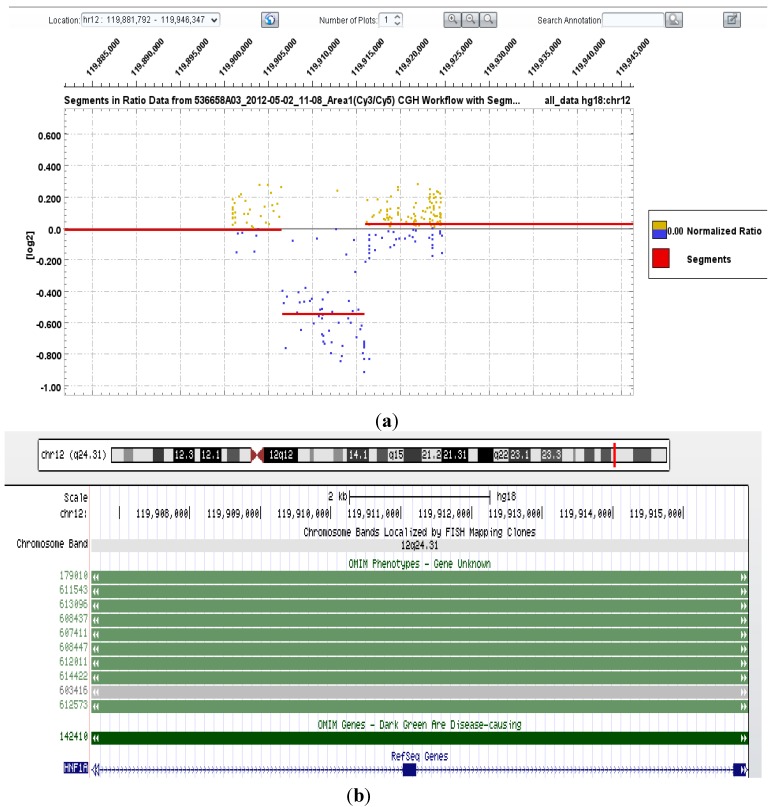
(**a**) DEVA software output showing copy number change (deletion; log2ratio: −0.5459) for probes localized to chr12:119906606-119915923 (hg18 co-ordinates), encompassing exons 2 and 3 of the *HNF1α* gene; (**b**) UCSC genome browser graphic output of chr12:119906606-119915923 (hg18 co-ordinates).

Two patients were referred for sequence and deletion/duplication analysis of the full MODY gene panel offered at our laboratory (genes listed above). No pathogenic mutations were detected on either assay in these patients. Patient 20, however, was referred for HNF1α gene analysis only. He was a 38 years old man with a history of multiple hepatic adenomas, requiring surgical resection, and a diabetic profile suggestive of MODY type 3. Biallelic inactivation of *HNF1α* has been reported to be an important event in the occurrence of liver adenoma [25]; partial or whole gene deletions are responsible for approximately 3% of cases of MODY type 3 [26]. Histological investigation of Patient 20’s resected hepatic tissue showed not only the three large lesions that had previously been noted on imaging, but also several hundred micro-adenomas. No pathogenic mutations were detected on sequence analysis of the *HNF1α* gene, but aCGH revealed an heterozygous deletion of exons 2–3 (inclusive; see Figure 2). This deletion removes the main part of the B domain and a portion of the homeodomain of the HNF1α protein, resulting in destabilization [27]. Mutation analysis of the affected hepatic tissue was not performed, but it is expected that somatic inactivation of the second HNF1α gene allele would be evident. 

## 4. Conclusions

Through the use of a gene-targeted CGH array for dosage analysis within the diagnostic environment, we have been able to confidently detect a spectrum of changes that would be invisible to sequence analysis: single exon, multiple exon and whole gene deletions/duplications. In addition, as a result of the high-density overlapping probes that tile the exons in our custom-designed array, we have found that large intra-exonic changes can also be detected (Patient 4 described above). 

The aCGH technique is robust and cost-effective, overcoming the problems associated with the use of expensive kits in the context of low sample throughput, and allowing for consolidation of previously separate gene-targeted dosage assays to a single validated technique. The cost-effectiveness is principally due to this ability to batch all samples received for deletion/duplication analysis, and to the fact that a separate assay does not need to be worked up for each gene, allowing analysis of a larger number of genes to be offered in-house and bringing more revenue into the laboratory. 

Furthermore, the aCGH process eliminates the risk of false positives that can occur as a result of polymorphisms under primer binding sites [20]. This risk is inherent in all PCR-based techniques, including the other dosage method most widely used by diagnostic laboratories, MLPA. To eliminate the occurrence of false positive results due to a one-off failure of hybridisation to a particular probe, each gene-focused probe on our custom-designed array is spotted in duplicate. In contrast to MLPA, aCGH allows the interrogation of intronic as well as exonic regions, allowing breakpoints to be mapped more accurately [20]. It can also be used to characterise some inversions and complex rearrangements, thereby offering a higher mutation detection rate than MLPA and other purely exon-focused dosage assays [28,29]. 

The disadvantages of the aCGH array approach described here are that it does not interrogate small-scale changes in deep intronic regions, nor rare and more complex rearrangements. These mutation events could, however, be detected by RNA analysis or whole genome sequencing. In the meantime, the combination of coding region sequence analysis and aCGH should detect the vast majority of pathogenic mutations known to be responsible for single gene disorders, thereby fulfilling the diagnostic needs of the clinical community.

During the latter stages of our study, we were informed that Nimblegen had ceased production of arrays. As a consequence, readers are directed to an alternative company, Agilent Technologies, which offers custom microarray designs that might serve as a suitable substitute.
